# Long-term kidney outcomes in patients with Kabuki syndrome

**DOI:** 10.1007/s00467-025-06815-0

**Published:** 2025-05-28

**Authors:** Seongjae Han, Hyeonju Lee, Peong Gang Park, Naye Choi, Yo Han Ahn, Jung Min Ko, Hee Gyung Kang

**Affiliations:** 1 Department of Pediatrics, Seoul National University Children’s Hospital, Seoul, Republic of Korea; 2 Department of Pediatrics, Ajou University School of Medicine, Suwon, Republic of Korea; 3 Department of Pediatrics, Seoul National University College of Medicine, Seoul, Republic of Korea; 4 Kidney Research Institute, Seoul National University Medical Research Center, Seoul, Republic of Korea

**Keywords:** Kabuki syndrome, Chronic kidney disease, Congenital anomalies of kidney and urinary tract, Nephrolithiasis

## Abstract

**Background:**

This study assessed the clinical features, prevalence of kidney and urinary manifestations, and progression of chronic kidney disease (CKD) in patients with Kabuki syndrome (KS).

**Methods:**

This retrospective cohort study enrolled patients with KS who visited a single tertiary center from 2003 to 2023.

**Results:**

Sixty-five patients (28 boys) were diagnosed with KS at a median age of 2.7 years (interquartile range [IQR] = 1.0–9.3) and followed until a median age of 9.4 years (IQR = 5.5–14.3). Genetic analysis identified *KMT2D* and *KDM6A* mutations in 59 and 3 patients, respectively. Congenital anomalies of the kidneys and urinary tract (CAKUT) were found in 21 of 62 patients (33.9%), whereas 7 of 62 patients (11.3%) patients had nephrolithiasis and/or nephrocalcinosis. Meanwhile, 19 of 56 patients (33.9%) progressed to CKD. CKD-free survival analysis illustrated that 25% and 50% of these patients progressed to CKD stage G2 at median ages of 5.8 and 24.6 years, respectively. Younger age at diagnosis and the presence of bilateral kidney anomalies were identified as significant predictors of CKD progression. CAKUT and cardiorenal syndrome were the leading causes of CKD.

**Conclusions:**

One-third of patients with KS exhibited various kidney or urinary abnormalities, and 34% progressed to CKD. Screening for kidney or urinary issues and regular follow-up of kidney function are essential for KS management.

**Graphical abstract:**

**Figure:**
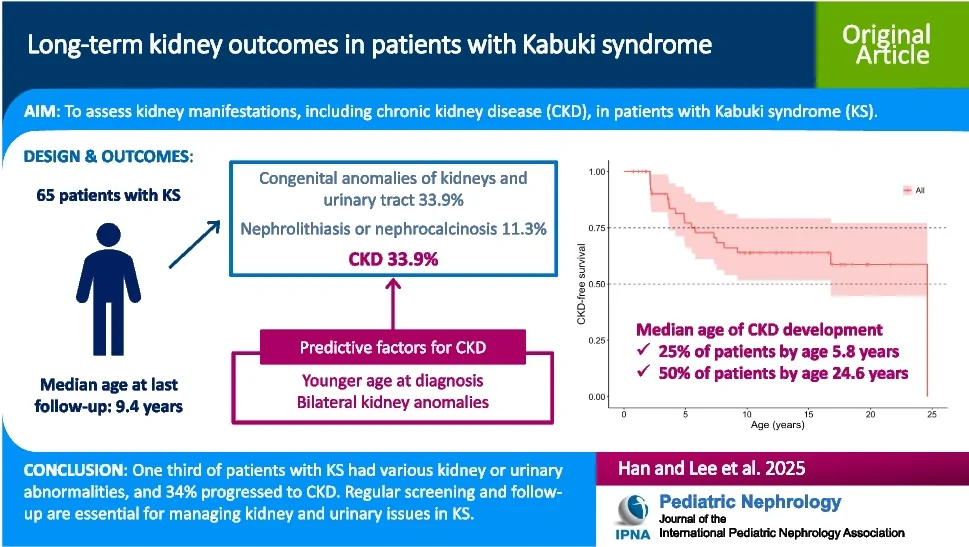
A higher resolution version of the Graphical abstract is available as Supplementary information

**Supplementary Information:**

The online version contains supplementary material available at https://doi.org/10.1007/s00467-025-06815-0.

## Introduction

Kabuki syndrome (KS; OMIM 147920) is a rare genetic syndrome caused by pathogenic variants in *KMT2D* (formerly *MLL2*; MIM #602,113) or *KDM6A* (MIM #30,012). Initially described independently in 1981 by Niikawa et al. and Kuroki et al., KS is characterized by five cardinal features: distinctive facial features, skeletal anomalies, dermatoglyphic abnormalities, intellectual disability, and short stature [1, 2]. The most prominent characteristic, the typical facial phenotype, includes long palpebral fissures with eversion of the lateral third of the lower eyelids and arched, broad eyebrows, giving the characteristic resemblance to the stage makeup used in Kabuki, a traditional Japanese theater form [3].

KS is associated with varied phenotypic features that affect multiple organ systems, including congenital heart defects and congenital anomalies of the kidneys and urinary tract (CAKUT). The incidence of CAKUT in patients with KS ranges 30–43%, being more common in individuals with *KMT2D* mutations [4–6]. CAKUT encompasses a broad spectrum of malformations, ranging from mild to severe, and it can lead to chronic kidney disease (CKD). There are reports of kidney failure requiring kidney transplantation caused by CAKUT and/or congenital heart defects in patients with KS [7–10]. However, few studies have specifically evaluated kidney function in patients with KS. This study assessed the clinical features, prevalence of kidney and urinary manifestations, and progression of CKD in patients with KS.

## Materials and methods

### Study design

This retrospective, single-center cohort study included all patients with KS who visited Seoul National University Children’s Hospital between August 2003 and August 2023. The diagnosis of KS was established according to the international consensus diagnostic criteria issued in 2019 [3]. Clinical, laboratory, and radiographic data were retrospectively collected from patients’ medical records and reviewed. The study was approved by the Seoul National University Hospital Institutional Review Board (IRB no. H-2309-043-1464).

### Definitions

The diagnoses of CAKUT, nephrocalcinosis, and nephrolithiasis were based on findings from kidney imaging studies, including ultrasonography and computed tomography. CAKUT was defined as abnormalities of kidney number, size, morphology, position, and/or abnormalities of outflow tract including the pelvis, ureter, or urinary bladder [11]. Bilateral kidney anomalies were defined as the presence of hypoplasia, dysplasia, or agenesis in both kidneys. Nephrocalcinosis was diagnosed and graded based on kidney ultrasonography as follows: grade 0, no echogenicity; grade 1, mild echogenicity around the medullary pyramid borders; grade 2, moderate echogenicity around and inside the pyramids; grade 3, severe echogenicity of all pyramids [12]. Hypercalciuria was defined as urine calcium/creatinine ratio exceeding 0.2 mg/mg. CKD stages G2 and G3 were defined by estimated glomerular filtration rates (eGFRs) of 60 to < 90 mL/min/1.73 m^2^ and 30 to < 60 mL/min/1.73 m^2^, respectively, in accordance with the GFR categories of the Kidney Disease: Improving Global Outcomes 2024 guidelines [13]. eGFR was calculated using the creatinine-based Chronic Kidney Disease in Children under 25 (CKiD U25) formula for children, adolescents, and young adults [14]. The diagnosis of congenital heart defects (CHD) was based on echocardiography. Left-sided obstructive CHD was defined as coarctation of aorta, bicuspid aortic valve, aortic or mitral stenosis, and hypoplastic left heart syndrome [15, 16].

### Genotype–phenotype correlation

For the causative gene-based analysis, patients were categorized into the *KMT2D* and *KDM6A* groups. To evaluate genotype–phenotype correlations within the *KMT2D* group, patients were further classified into predicted loss-of-function (pLoF) and non-pLoF variant groups based on variant type. Pathogenic nonsense, frameshift, splicing variants, and deletions were classified as pLoF variants, whereas missense and in-frame variants were classified as non-pLoF variants. For the variant location-based analysis, the pLoF variant group was further subdivided into the C-terminus group, which included variants located in or after exon 39 of *KMT2D*, and the non-C-terminus group, which included variants located before exon 39 of *KMT2D* [17]. Since all non-pLoF variants were located in or after exon 39 of *KMT2D*, location-based analysis was not applicable in the non-pLoF variant group.

### Statistical analysis

Statistical analyses were performed using IBM® SPSS® Statistics 29.0 (IBM, Armonk, NY, USA) and R version 4.2.2. Data were reported as medians and interquartile ranges (IQRs). Categorical variables were analyzed using Fisher’s exact test, whereas continuous variables were compared using the Mann–Whitney *U* test. CKD-free survival was evaluated using Kaplan–Meier survival analysis. Cox proportional hazards regression was employed to identify predictive factors for CKD-free survival. Variables with *P* < 0.200 in univariate analysis were included in the multivariate Cox proportional hazards model. Significance was indicated by *P* < 0.05.

## Results

### Patient characteristics

In total, 65 children with KS of Korean descent, including 28 boys, were enrolled in this study (Table 1). The median age at diagnosis was 2.7 years (IQR = 1.0–9.3), and the median age at the last follow-up was 9.4 years (IQR = 5.5–14.3). All patients underwent genetic testing, including Sanger sequencing, targeted exome sequencing, whole exome sequencing, multiplex ligation-dependent probe amplification, or whole genome sequencing (Supplementary Table 1). Most patients (90.8%) had *KMT2D* mutations, whereas three patients each harbored *KDM6A* mutations and unidentified mutations. The *KMT2D* mutations consisted of various types, including frameshift mutations (*n* = 23), nonsense mutations (*n* = 21), missense mutations (*n* = 8), splicing site mutations (*n* = 6), and deletion (*n* = 1).

**Table 1:** Characteristics of patients with Kabuki syndrome

Characteristics	Total (*n* = 65)
Sex	
Male	28 (43.1%)
Female	37 (56.9%)
Causative gene	
* KMT2D*	59 (90.8%)
Frameshift	23 (35.4%)
Nonsense	21 (32.3%)
Missense	8 (12.3%)
Splicing site	6 (9.2%)
Exonic deletion	1 (1.5%)
* KDM6A*	3 (4.6%)
Not identified	3 (4.6%)
Median age at diagnosis, years	2.7 (IQR 1.0–9.3)
Median age at the last follow up, years	9.4 (IQR 5.5–14.3)
CAKUT	21/62 (33.9%)
Horseshoe kidney	8 (12.9%)
Ectopic kidney	7 (11.3%)
Hypoplastic kidney	6 (9.7%)
Multicystic dysplastic kidney	4 (6.5%)
Duplex kidney	2 (3.2%)
Unilateral renal agenesis	1 (1.6%)
Ureteropelvic junction obstruction	1 (1.6%)
Nephrolithiasis and/or nephrocalcinosis	7/62 (11.3%)
Kidney and/or ureter stone	5 (8.1%)
Nephrocalcinosis	4 (6.5%)

*IQR* interquartile ranges, *CAKUT* congenital anomalies of kidneys and urinary tract

### Kidney manifestations

Kidney and urinary system was radiologically evaluated in 62 patients (95.4%), revealing CAKUT in 21 patients (33.9%). The specific anomalies included horseshoe kidney (*n* = 8), ectopic kidney (*n* = 7), hypoplastic kidney (*n* = 6), multicystic dysplastic kidney (*n* = 4), duplex kidney (*n* = 2), unilateral renal agenesis (*n* = 1), and ureteropelvic junction obstruction (*n* = 1; Table 1).

Nephrolithiasis and/or nephrocalcinosis were identified in 7 of 62 patients (11.3%) at a median age of 5.5 years (IQR = 3.6–13.5). Two patients were diagnosed with both nephrocalcinosis and kidney stones. Nephrocalcinosis was localized in the medullary region in all four affected patients and was classified as grade 1. Among these patients, hypercalciuria was detected in all six patients who underwent urinary testing related to stone formation. One patient with hypoplastic left heart syndrome had been prescribed loop diuretics, a drug known to cause hypercalciuria. Two patients required surgical intervention for stone removal, and they subsequently received potassium citrate. Stone analysis revealed calcium oxalate monohydrate and calcium phosphate compositions. Another patient was treated with hydrochlorothiazide and potassium citrate. The remaining four patients did not experience progression of nephrolithiasis and/or nephrocalcinosis without pharmacological intervention. No patients with nephrolithiasis experienced chronic obstruction or recurrent urinary tract infections.

### Kidney function and CKD progression

**Fig. 1:**
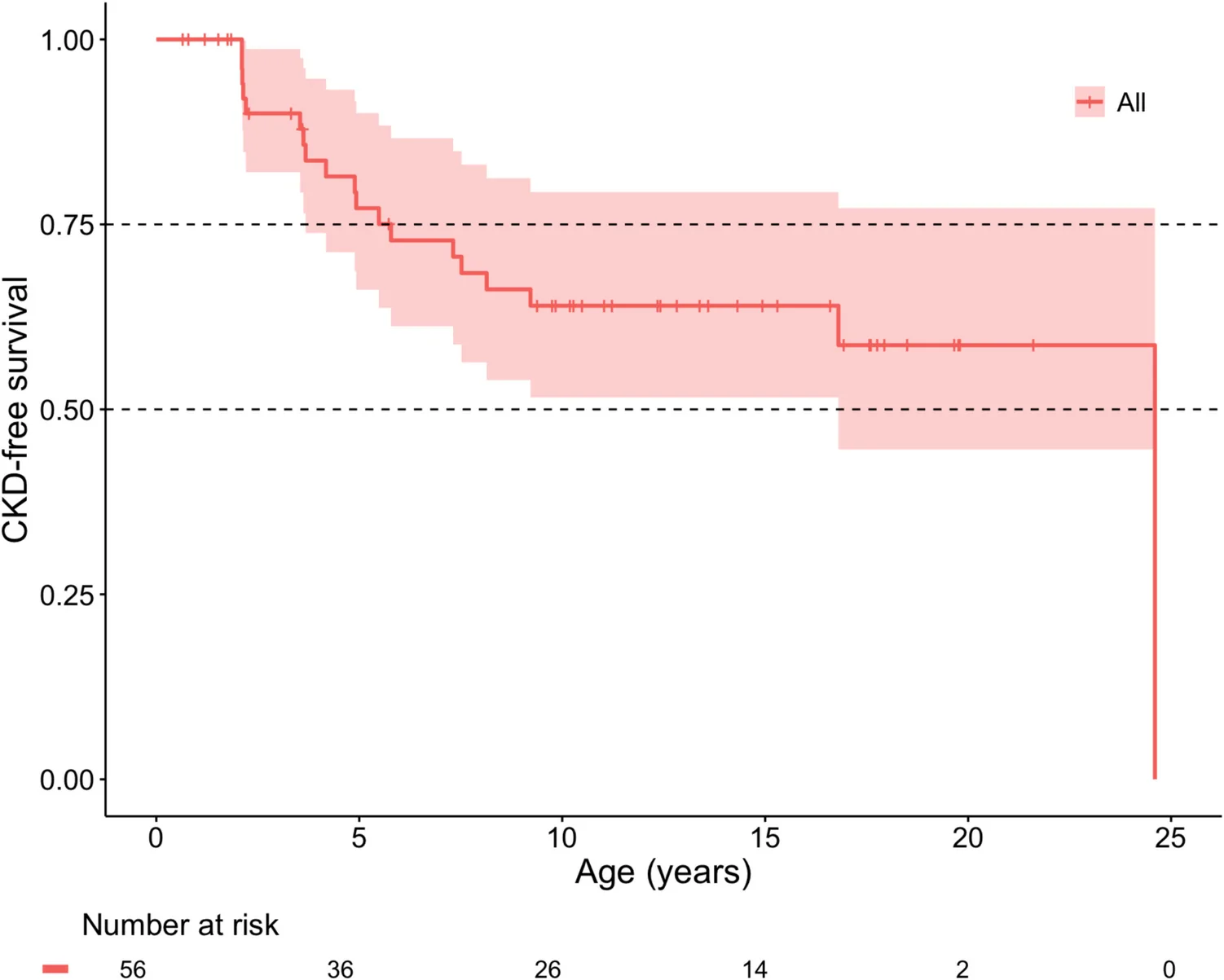
Chronic kidney disease-free survival of patients with Kabuki syndrome. The solid line represents the survival curve estimated using Kaplan–Meier analysis, with shaded areas indicating the 95% confidence intervals

**Fig. 2:**
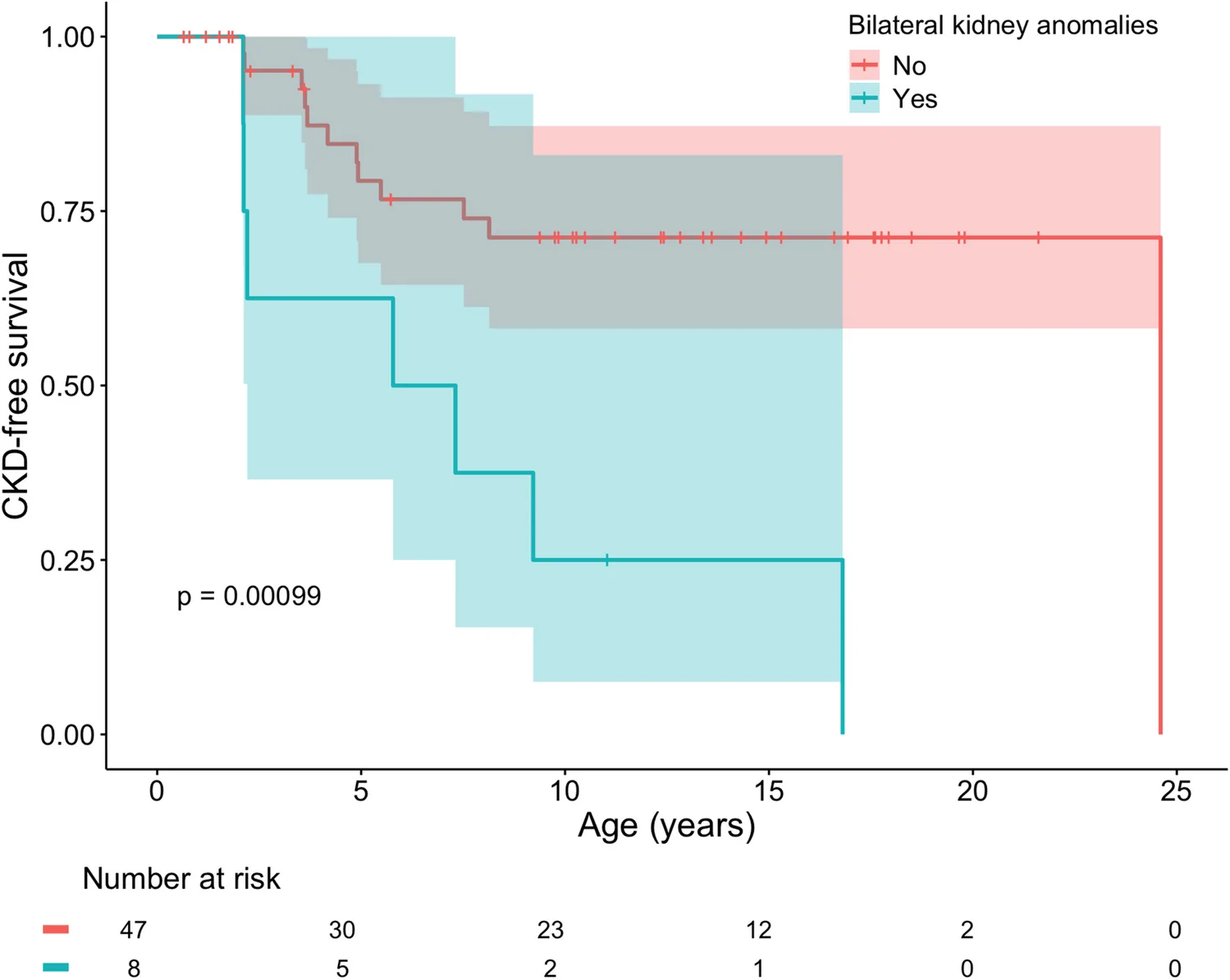
Chronic kidney disease-free survival according to the presence of bilateral kidney anomalies. The solid line represents the survival curve estimated using Kaplan–Meier analysis, with shaded areas indicating the 95% confidence intervals

Serum creatinine levels were measured in 56 patients (86.2%), who were subsequently followed until a median age of 7.5 years (IQR = 3.7–13.5) with a median of 7 creatinine measurements (IQR = 1–13.5). Among these patients, 33.9% (*n* = 19) experienced CKD progression, including progression to stage G2 in 16 patients and stage G3 in 3 patients. Kaplan–Meier survival analysis estimated that 25% and 50% of patients developed CKD G2 at median ages of 5.8 (95% confidence interval [CI] = 3.7–not available) and 24.6 years (95% CI = 16.8–not available), respectively (Fig. 1). To identify predictive factors for CKD progression, Cox proportional hazard models were analyzed, incorporating demographic data, genetic variant types, and the presence and subtypes of CAKUT and CHD. Multivariate Cox proportional hazards analysis demonstrated that younger age at diagnosis and the presence of bilateral kidney anomalies were significant predictive factors for progression to CKD G2 (Table 2). Kaplan–Meier analysis demonstrated a significant difference in CKD-free survival based on the presence of bilateral kidney anomalies (Fig. 2).

**Table 2:** Predictive factors for chronic kidney disease progression in patients with Kabuki syndrome

Variables	Univariate		Multivariate	
	Hazard ratio (95% CI)	*P* value	Hazard ratio (95% CI)	*P* value
Male sex	1.101 (0.433–2.800)	0.840	–	–
Age at diagnosis	0.741 (0.625–0.880)	< 0.001	0.746 (0.609–0.914)	0.005
Causative gene, *KMT2D*	2.858 (0.379–21.550)	0.308	–	–
*KMT2D* genotype, pLoF variants	1.792 (0.238–13.520)	0.572	–	–
Location of pLoF variants in *KMT2D*, C-terminus	0.839 (0.303–2.325)	0.736	–	–
CAKUT	3.244 (1.251–8.415)	0.016	1.156 (0.315–4.240)	0.827
Bilateral kidney anomalies	4.337 (1.675–11.230)	0.003	3.896 (1.118–13.577)	0.033
Nephrolithiasis and/or nephrocalcinosis	0.722 (0.165–3.160)	0.666	–	–
CHD	2.138 (0.753–6.072)	0.154	0.814 (0.244–2.716)	0.738
Operative treatment for CHD	1.008 (0.378–2.691)	0.987	–	–
Left-sided obstructive CHD	1.356 (0.392–4.691)	0.630	–	–

*CI* confidence intervals, *pLoF* predicted loss-of-function, *CAKUT* congenital anomalies of kidney and urinary tract, *CHD* congenital heart defect

The characteristics and associated pathologies of the 19 patients with CKD are detailed in Table 3. Among the causes of CKD, seven patients had CAKUT, two had cardiorenal syndrome attributable to a significant congenital heart defect, and four had both conditions. CKD was associated with perinatal factors, including prematurity, low birth weight, or hypoxic injury, in three patients. One patient (ID 40 in Table 3) experienced two episodes of urinary tract infection within a cyst due to infundibular stenosis in the upper pole of a right duplicated kidney and subsequently underwent right partial nephrectomy. No other patients had chronic obstruction or recurrent urinary tract infections contributing to CKD development.

**Table 3:** Clinical information of patients who developed CKD (*n* = 19)

ID	Sex	Gene	Onset age of CKD G2 (years)	Cause of CKD	Kidney manifestations	Congenital heart defects
1	M	*KMT2D*	8.1	CAKUT	Rt renal agenesis	–
3	F	*KMT2D*	4.9	Prematurity	–	ASD
7	M	*KMT2D*	2.1	CAKUT and nephrocalcinosis with kidney stone	Bilateral dysplastic kidneys	ASD and small PDA
8	F	*KDM6A*	24.6	Nephrotic syndrome	–	–
9	F	*KMT2D*	2.1	CAKUT	Bilateral hypodysplastic kidneys	Small VSD
10	F	*KMT2D*	2.1	Cardiorenal syndrome	–	HLHS
14	F	*KMT2D*	5.5	CAKUT and cardiorenal syndrome	Horseshoe kidney	CoA and VSD
15	F	Not identified	3.5	AKI from perinatal hypoxic injury	–	PFO
16	M	*KMT2D*	9.2	CAKUT and cardiorenal syndrome	Lt hypoplastic kidney with rt ectopic kidney	VSD, ASD, and PDA
17	F	*KMT2D*	5.8	CAKUT	Bilateral ectopic kidneys with rt hypoplastic kidney	ASD
20	M	*KMT2D*	7.3	CAKUT	Bilateral hypoplastic kidneys	–
25	F	*KMT2D*	7.5	CAKUT and cardiorenal syndrome	Horseshoe kidney	large ASD
35	M	*KMT2D*	4.2	Unknown	–	–
36	M	*KMT2D*	2.1	Cardiorenal syndrome	–	VSD and ASD
40	F	*KMT2D*	16.8	CAKUT	Lt ectopic kidney and rt duplicated kidney (s/p rt partial nephrectomy)	DOMV and mild MS
46	F	*KMT2D*	3.7	SGA	–	–
52	F	*KMT2D*	3.6	CAKUT	Horseshoe kidney	–
62	M	*KMT2D*	4.9	Unknown	–	–
64	M	*KMT2D*	2.2	CAKUT and cardiorenal syndrome	Crossed fused ectopic kidney with dysplasia	CoA and VSD

*G*, glomerular filtration rate category; *M*, male; *F*, female; *Rt*, right; *Lt*, left; *CKD*, chronic kidney disease; *AKI*, acute kidney injury; *CAKUT*, congenital anomalies of kidney and urinary tract; *ASD*, atrial septal defect; *VSD*, ventricular septal defect; *PDA*, patent ductus arteriosus; *CoA*, coarctation of aorta; *HLHS*, hypoplastic left heart syndrome; *PFO*, patent foramen ovale; *DOMV*, double orifice mitral valve; *MS*, mitral stenosis; *SGA*, small for gestational age; *s/p*, status post

The three patients who progressed to CKD stage G3 included one boy (ID 7 in Table 3) with bilateral dysplastic kidneys and nephrocalcinosis who was treated with thiazide and potassium citrate for hypercalciuria. He progressed to stage G3 at 6.1 years old. A girl (ID 10 in Table 3) with hypoplastic left heart syndrome developed stage G3 CKD at 4.4 years old. The third patient was a girl (ID 15 in Table 3) who experienced meconium aspiration syndrome and hypoxic insult at birth, which was complicated by acute kidney injury. This acute kidney injury contributed to the progression of CKD at 3.5 years old.

### Genotype–phenotype correlation analysis

Patients with *KMT2D* mutations were diagnosed with KS at an earlier age than those with *KDM6A* mutations; however, no significant differences in kidney manifestations were observed between the two groups (Table 4). Among patients with *KMT2D* mutations, no statistically significant differences were found between the non-pLoF and pLoF variant groups in terms of sex ratio, age at diagnosis, or the prevalence of CAKUT, nephrolithiasis/nephrocalcinosis, and CKD. In the location-based analysis of patients with pLoF variants in *KMT2D*, the C-terminus group exhibited a significantly higher prevalence of nephrolithiasis and/or nephrocalcinosis compared to the non-C-terminus group. Kaplan–Meier survival analysis showed no significant differences in CKD-free survival based on the causative gene of KS, *KMT2D* genotype, or the location of pLoF variants in *KMT2D* (Supplementary Fig. 1).

**Table 4:** Genotype–phenotype correlations in patients with Kabuki syndrome for kidney and urinary manifestations

Characteristics	*KDM6A* (*n* = 3)	*KMT2D* (*n* = 59)	*P* value	*KMT2D* (*n* = 59)					
				Non-pLoF (*n* = 8)	pLoF (*n* = 51)				
					Total (*n* = 51)	Non-C-terminus (*n* = 32)	C-terminus (* n* = 19)	*P* value^a^	*P* value^b^
Sex, male:female	1:2	26:33	1.000	5:3	21:30	11:21	10:9	0.484	0.247
Age of KS diagnosis, years	11.6 (10.8–16.7)	2.2 (0.7–8.0)	0.025	4.8 (1.9–8.4)	2.1 (0.6–8.0)	1.8 (0.6–5.2)	4.2 (0.6–10.7)	0.303	0.328
CAKUT	0/2 (0.0)	20/57 (36.8)	0.534	1/7 (14.3)	20/50 (40.0)	14/31 (45.2)	6/19 (31.6)	0.243	0.387
Nephrolithiasis and/or nephrocalcinosis	0/2 (0.0)	6/57 (10.5)	1.000	0/7 (0.0)	6/50 (12.0)	1/31 (3.2)	5/19 (26.3)	1.000	0.024
CKD G2	1/3 (33.3)	17/50 (34.0)	1.000	1/6 (16.7)	16/44 (36.4)	10/26 (38.5)	6/18 (33.3)	0.650	0.761

Values are presented as numbers (%) or median (interquartile range)

*pLoF* predicted loss of function, *KS* Kabuki syndrome, *CAKUT* congenital anomalies of kidneys and urinary tract, *CKD* chronic kidney disease, *G* glomerular filtration rate category

^a^Non-pLoF vs. pLoF

^b^Non-C-terminus of pLoF vs. C-terminus of pLoF

## Discussion

Our study demonstrated that patients with KS exhibit various kidney manifestations, including a CKD progression rate of one-third. The frequency of CAKUT observed in this cohort was consistent with that in previous studies [5, 6, 18]. To our knowledge, this is the first report examining the long-term outcomes of kidney function and the prevalence of nephrolithiasis/nephrocalcinosis in patients with KS.

Whereas previous reports on patients with KS and kidney failure provided insights into the etiologies of CKD in this population [5, 7–10, 19, 20], no follow-up analyses assessed the time course of CKD development. Our study addressed this gap by using CKD-free survival analysis to estimate the time until CKD onset in patients with KS. Through a literature review, we identified seven patients with KS who developed kidney failure. Five of these patients underwent kidney transplantation, one progressed to stage 4 CKD, and one died of kidney failure and pulmonary hypertension (Supplementary Table 2) [5, 7–10, 19, 20]. In six of these patients, CAKUT and/or congenital heart defects contributed to CKD progression, consistent with our findings. Additionally, some of our patients had perinatal factors, including prematurity, small for gestational age, and hypoxic injury at birth, as contributing causes of CKD. In this study, younger age at diagnosis and the presence of bilateral kidney anomalies were associated with CKD progression. Bilateral involvement of kidney anomalies has been shown to influence CKD progression in patients with CAKUT [21, 22], while an earlier age at diagnosis may reflect more severe clinical manifestations of KS. The KDIGO guideline recommends screening individuals at risk for CKD, including those with CAKUT, recurrent kidney stones, and known genetic variants associated with CKD, through both urine albumin measurement and GFR assessment [13]. To mitigate kidney function deterioration, early detection and intervention are crucial. Clinicians should focus on managing modifiable risk factors for CKD progression, such as hypertension and proteinuria, while also promoting lifestyle modifications, including regular physical activity, maintaining an optimal weight, and adhering to a kidney-protective diet, particularly in young children and those with bilateral kidney anomalies [13].

A prior single-center cohort study reported that 3 of 32 patients (9.4%) with nephrolithiasis/nephrocalcinosis had KS caused by *KMT2D* mutations [23]. No previous studies reported the prevalence of nephrolithiasis/nephrocalcinosis specifically in patients with KS and *KMT2D* mutations. One study indicated that 2 of 61 patients with *KDM6A* mutations had nephrolithiasis/nephrocalcinosis [18]. Although the precise mechanisms underlying stone formation in patients with KS remain unclear, our cases of nephrolithiasis/nephrocalcinosis were associated with hypercalciuria, which can lead to calcium crystal deposition in the kidneys. Given that symptomatic stone formation can necessitate surgical intervention in children, clinicians should conduct urinalysis and kidney imaging to facilitate the early detection and management of hypercalciuria and nephrolithiasis in patients with KS. For patients with hypercalciuria, management should include dietary modifications (optimized sodium, protein, and potassium intake), adequate hydration, and pharmacologic interventions such as thiazide diuretics and potassium citrate to reduce urinary calcium excretion and prevent stone formation [24].

Previous research suggested that patients with KS caused by *KMT2D* mutations are more likely to have kidney and urinary abnormalities than those without these mutations [5, 6]. Regarding *KDM6A*, one study reported a lower prevalence of kidney and urinary tract malformations in patients with KS caused by *KDM6A* mutations than that in the overall KS population [18]. In our cohort, no significant differences in kidney and urinary manifestations were observed between the *KMT2D* and *KDM6A* groups. However, given the limited number of patients with *KDM6A* mutations, these findings may not be conclusive. We found no correlation between kidney phenotypes and genotypes in patients with *KMT2D* mutations, consistent with previous studies [5, 6]. A prior study reported all cases with visceral anomalies, including genitourinary abnormalities, were observed in the protein-truncating variant group; however, the sample size of the non-truncating group was small (*n* = 4) [25]. In our location-based analysis of pLoF variants in *KMT2D*, nephrocalcinosis and/or nephrolithiasis were more prevalent in patients with variants located towards the C-terminus. The *KMT2D* gene’s C-terminal region plays a critical role in its function as a histone H3 K4 methyltransferase, influencing gene expression by modulating chromatin structure [17]. Although our findings are limited by the inability to conduct location-based analysis for non-pLoF mutations and the lack of differences in other kidney manifestations, this suggests that pLoF mutations near the C-terminus may be associated with nephrocalcinosis/nephrolithiasis. Similarly, a multicenter cohort study observed greater rates of severe eye involvement in nonsense variants near the C-termini of *KMT2D* and *KDM6A*, whereas frameshift variants were not associated with structural ocular abnormalities [17]. However, in our study, when the analysis was restricted to nonsense variants, no significant differences in kidney and urinary manifestations were observed based on variant location (Supplementary Table 3). While a previous study suggested no association between the location of genetic mutations and kidney or urinary manifestations [5], our study is the first to specifically evaluate nephrocalcinosis and nephrolithiasis in this context. Given the limited evidence and lack of clear mechanistic explanation for this association, further studies are needed to validate these findings.

This study had several limitations. First, data collection and analysis were conducted retrospectively. Kidney function assessments and imaging studies were not performed according to standardized protocols, potentially leading to an underestimation of kidney manifestations. Second, the follow-up period was limited, with most patients in our cohort still in their teens, providing limited information on kidney function in adults with KS. Long-term monitoring of kidney function in patients with KS extending into adulthood is essential for both future research and patient care, particularly for the early detection and management of CKD.

In conclusion, more than 30% of patients with KS exhibited kidney or urinary involvement, and 34% progressed to CKD. Therefore, screening for kidney or urinary issues and regular follow-up of kidney function are essential for managing patients with KS. A review article has recommended initial evaluation and organ-specific health surveillance in patients with KS, including baseline kidney ultrasound to assess CAKUT [26]. We recommend blood and urine tests every 2–3 years for kidney function surveillance, along with endocrinologic and immunologic assessments [26]. Additionally, if CKD or nephrolithiasis/nephrocalcinosis is detected, a tailored follow-up schedule should be implemented.

## Supplementary information

Below is the link to the electronic supplementary material.

Graphical abstract (PPTX 165 KB)

ESM 2 (DOCX 33.6 KB)

## Data Availability

The data that support the findings of this study are not openly available due to reasons of sensitivity and are available from the corresponding author upon reasonable request.
