# Supplementary material for: Long-term kidney outcomes in patients with Kabuki syndrome
Source: Pediatr Nephrol. 2025 May 28;40(10):3101–9. doi: 10.1007/s00467-025-06815-0 (PMC12402012; doi:10.1007/s00467-025-06815-0)
Supplement: Supplementary file 1 — Graphical abstract (PPTX 165 KB) [file 467_2025_6815_MOESM1_ESM.pptx]

## Slide 1
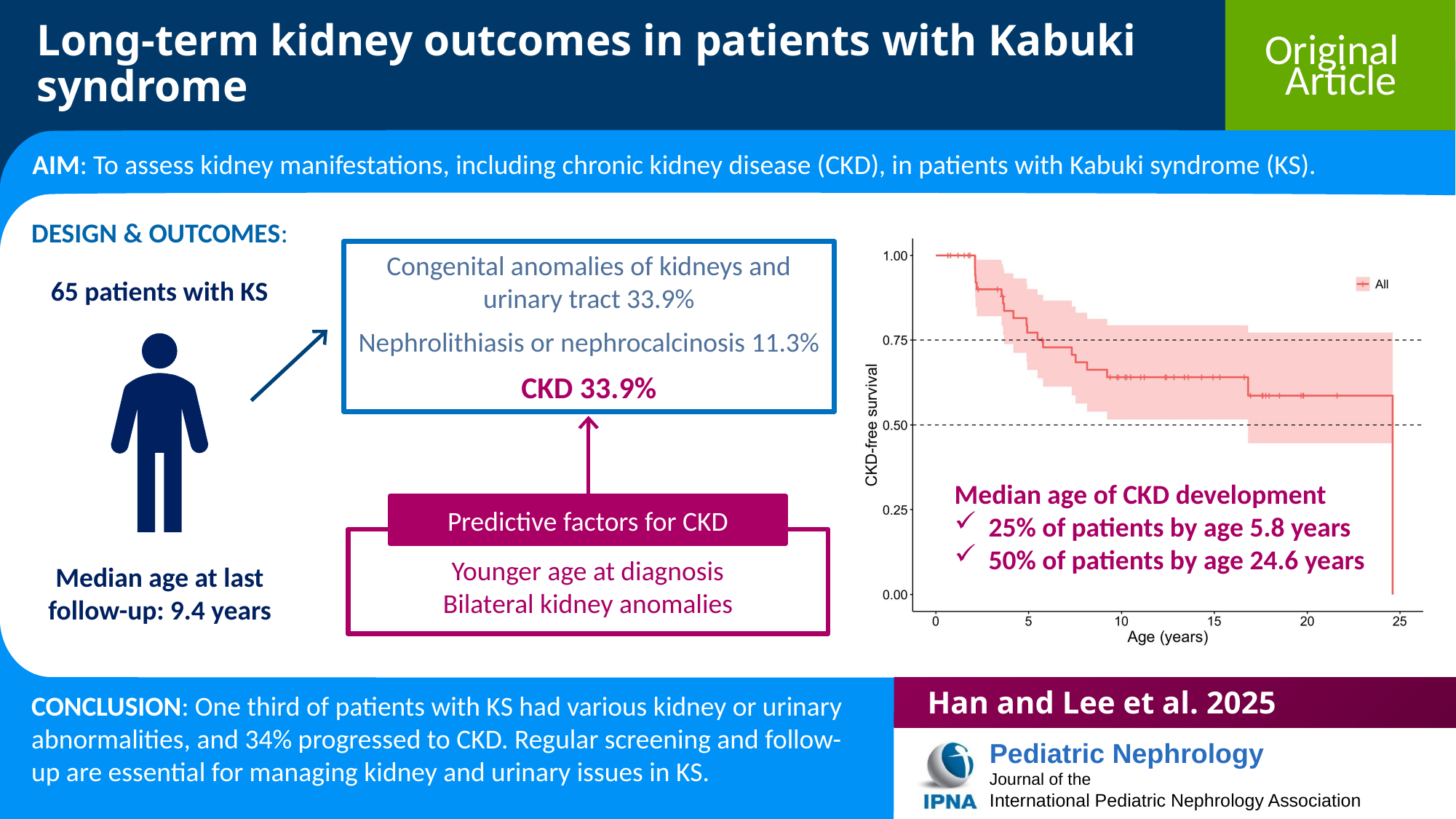

Long-term kidney outcomes in patients with Kabuki syndrome
AIM: To assess kidney manifestations, including chronic kidney disease (CKD), in patients with Kabuki syndrome (KS).
DESIGN & OUTCOMES:
Median age of CKD development
25% of patients by age 5.8 years
50% of patients by age 24.6 years
Congenital anomalies of kidneys and urinary tract 33.9%
Nephrolithiasis or nephrocalcinosis 11.3%
CKD 33.9%
65 patients with KS
Predictive factors for CKD
Younger age at diagnosis
Bilateral kidney anomalies
Median age at last follow-up: 9.4 years
Han and Lee et al. 2025
CONCLUSION: One third of patients with KS had various kidney or urinary abnormalities, and 34% progressed to CKD. Regular screening and follow-up are essential for managing kidney and urinary issues in KS.
