# Supplementary material for: Long-term kidney outcomes in patients with Kabuki syndrome
Source: Pediatr Nephrol. 2025 May 28;40(10):3101–9. doi: 10.1007/s00467-025-06815-0 (PMC12402012; doi:10.1007/s00467-025-06815-0)
Supplement: Supplementary file 2 — ESM 2 (DOCX 33.6 KB) [file 467_2025_6815_MOESM2_ESM.docx]

Supplementary Table 1. Genetic diagnosis of patients with Kabuki syndrome

| ID | Sex | Gene | cDNA change | Protein change | Pathogenicity | Genetic test |
| --- | --- | --- | --- | --- | --- | --- |
| 1 | M | *KMT2D* | c.9355A>T | p.Lys3119* | LP | TES |
| 2 | F | *KMT2D* | c.3678_3679insT | p.Leu1227Serfs*23 | LP | TES |
| 3 | F | *KMT2D* | c.3808dup | p.Ser1270Phefs*16 | LP | TES |
| 4 | F | *KMT2D* | c.15142C>T | p.Arg5048Cys | P | WES |
| 5 | F | *KMT2D* | c.13201C>T | p.Gln4401* | LP | TES |
| 6 | M | *KMT2D* | c.8464C>T | p.Gln2822* | LP | TES |
| 7 | M | *KMT2D* | c.16342C>T | p.Arg5448* | P | TES |
| 8 | F | *KDM6A* | c.286_289del | p.Phe96Valfs*3 | LP | WES |
| 9 | F | *KMT2D* | c.7411C>T | p.Arg2471* | P | TES |
| 10 | F | *KMT2D* | c.15686del | p.Arg5229Profs*14 | LP | TES |
| 11 | F | *KMT2D* | c.7325del | p.Pro2442Lfs*43 | P | TES |
| 12 | M | *KMT2D* | c.15640C>T | p.Arg5214Cys | P | Sanger |
| 13 | M | *KMT2D* | c.16052G>A | p.Arg5351Gln | P | TES |
| 14 | F | *KMT2D* | c.6595del | p.Tyr2199llefs*65 | P | TES |
| 15 | F | Not identified | NA | NA | NA | TES |
| 16 | M | *KMT2D* | c.7007del | p.Ser2336Phefs*3 | LP | TES |
| 17 | F | *KMT2D* | c.10357C>T | p.Gln3453* | LP | TES |
| 18 | F | *KMT2D* | c.6992del | p.Leus2331Argfs*8 | P | TES |
| 19 | M | *KMT2D* | c.11833C>T | p.Gln3945* | LP | TES |
| 20 | M | *KMT2D* | c.11944C>T | p.Arg3982* | P | TES |
| 21 | M | *KMT2D* | c.16052+1G>A | NA | LP | TES |
| 22 | F | *KMT2D* | c.15217C>T | p.Gln5073* | P | TES |
| 23 | M | *KMT2D* | c.15461G>A | p.Arg5154Gln | P | TES |
| 24 | F | *KMT2D* | c.11737C>T | p.Gln3913* | LP | TES |
| 25 | F | *KMT2D* | c.13285C>T | p.Gln4429* | LP | TES |
| 26 | M | *KMT2D* | c.14945G>A | p.Trp4982* | P | Sanger |
| 27 | F | *KMT2D* | c.11515C>T | p.Gln3839* | P | TES |
| 28 | M | *KMT2D* | c.10629_10630del | p.Ala3544Glnfs*11 | LP | TES |
| 29 | F | *KMT2D* | c.8200C>T | p.Arg2734* | P | WGS |
| 30 | F | *KMT2D* | c.4135_4136del | p.Met1379Valfs*52 | P | TES |
| 31 | M | *KMT2D* | c.12592C>T | p.Arg4198* | P | TES |
| 32 | M | *KMT2D* | c.15088C>T | p.Arg5030Cys | P | TES |
| 33 | F | *KMT2D* | c.12021del | p.Gln4007Hisfs*15 | LP | TES |
| 34 | F | *KMT2D* | c.7710del | p.Thr2571Profs*12 | LP | TES |
| 35 | M | *KMT2D* | c.303_304insG | p.Ser102Glufs*6 | P | TES |
| 36 | M | *KMT2D* | c.7099_7102del | p.Asp2367Serfs*16 | LP | TES |
| 37 | F | *KMT2D* | c.16159C>T | p.Gln5387* | LP | TES |
| 38 | F | *KDM6A* | c.3876_3878del and 3878+1del | Exon 26 skipping | P | TES |
| 39 | M | *KMT2D* | c.10356-47A>G | NA | LP | TES |
| 40 | F | *KMT2D* | c.4135_4136del | p.Met1379Valfs*52 | P | TES |
| 41 | F | *KMT2D* | c.176+1G>C | NA | LP | TES |
| 42 | F | *KMT2D* | c.646G>T | p.Glu216* | LP | TES |
| 43 | M | *KMT2D* | c.176+1G>T | NA | LP | Sanger |
| 44 | M | *KDM6A* | c.974G>C | p.Gly325Ala | LP | TES |
| 45 | F | *KMT2D* | c.6553del | p.Leu2185Trpfs*79 | LP | TES |
| 46 | F | *KMT2D* | c.3907-2A>C | NA | LP | TES |
| 47 | F | *KMT2D* | exonic deletion including exon 10 | NA | P | MLPA |
| 48 | M | *KMT2D* | c.241G>T | p.Glu81* | LP | TES |
| 49 | F | *KMT2D* | c.5775_5776insA | p.Leu1926Thrfs*31 | P | TES |
| 50 | F | *KMT2D* | c.15142C>T | pArg5048Cys | P | TES |
| 51 | M | *KMT2D* | c.9961C>T | p.Arg3321* | P | TES |
| 52 | F | *KMT2D* | c.16261A>C | p.Thr5421Pro | LP | TES |
| 53 | M | *KMT2D* | c.7200del | p.Arg2401Alafs*25 | P | TES |
| 54 | M | *KMT2D* | c.16294C>T | p.Arg5432Trp | P | TES |
| 55 | M | *KMT2D* | c.13531-1delG | NA | LP | Sanger |
| 56 | F | *KMT2D* | c.5775_5776insA | p.Leu1926Thrfs*31 | P | Sanger |
| 57 | F | Not identified | NA | NA | NA | TES |
| 58 | M | Not identified | NA | NA | NA | TES |
| 59 | F | *KMT2D* | c.15061C>T | p.Arg5021* | P | WES |
| 60 | F | *KMT2D* | c.7618C>T | p.Gln2540* | LP | Sanger |
| 61 | F | *KMT2D* | c.5778_5782+7delinsGAAGTGGGAG | p.Gln1927Lysfs*122 | LP | Sanger |
| 62 | M | *KMT2D* | c.12835dup | p.Ala4279Glyfs*55 | LP | TES |
| 63 | M | *KMT2D* | c.2232_2233insG | p.Pro745Alafs*4 | P | TES |
| 64 | M | *KMT2D* | c.12994_12995insT | p.Thr4332Ilefs*2 | P | TES |
| 65 | F | *KMT2D* | c.6827dup | p.Pro2277Thrfs*22 | LP | Sanger |

*M*, male; *F*, female; *P*, pathogenic; *LP*, likely pathogenic; *MLPA*, multiplex ligation-dependent probe amplification; *NA*, not available; *TES*, targeted exome sequencing; *WES*, whole exome sequencing; *WGS*, whole genome sequencing

Supplementary Table 2. Previously reported cases of kidney failure in patients with Kabuki syndrome

| **Reference** | **Kidney function** | **Potential causes of kidney failure** |
| --- | --- | --- |
| Ewart-Toland et al., 1998 [7] | Kidney TPL at 6 years of age | Bilateral dysplastic kidneys |
| Armstrong et al., 2005 [19] | Died from kidney failure and pulmonary hypertension at 5 years of age | Unspecified |
| Hamdi Kamel et al., 2006 [8] | Kidney TPL at 14 years of age | Dysplastic and non-functioning left kidney with right refluxing megaureter  Coarctation of aorta  Birth asphyxia secondary to meconium aspiration |
| Courcet et al., 2013 [5] | Kidney failure in neonatal period  eGFR of 18mL/min/1.73 m^2^ at 2.5 years old | Left renal agenesis and severe right hypoplastic kidney with poor cortico-medullary differentiation  Hypoplastic aortic arch with bicuspid aortic valve |
| Kohei et al., 2016 [9] | Kidney TPL at 12 years of age | Bilateral kidney hypoplasia |
| Kerr et al., 2017 [10] | Kidney TPL at 13 years of age | Coarctation of aorta |
| Merdler-Rabinowicz et al., 2020 [20] | Kidney TPL at 20 years of age | Renal agenesis |

*TPL*, transplantation; *eGFR*, estimated glomerular filtration rate

Supplementary Table 3. Comparison for kidney manifestations according to the location of variants among patients with nonsense variants in *KMT2D*

| Characteristics | Non-C-terminus (n=9) | C-terminus (n=12) | *P* value |
| --- | --- | --- | --- |
| Sex, male:female | 4:5 | 5:7 | 1.000 |
| Age of KS diagnosis, years | 2.7 (0.8–9.2) | 8.0 (2.4–13.2) | 0.193 |
| CAKUT | 6/9 (66.7) | 5/12 (41.7) | 0.387 |
| Nephrolithiasis and/or  Nephrocalcinosis | 0/9 (0) | 3/12 (25.0) | 0.229 |
| CKD G2 | 3/7 (42.9) | 3/12 (25.0) | 0.617 |

Values are presented as numbers (%) or median (interquartile range).

*KS*, Kabuki syndrome; *CAKUT*, congenital anomalies of kidneys and urinary tract; *CKD*, chronic kidney disease; *G*, glomerular filtration rate category

Supplementary Figure legends

Supplementary Figure 1. Chronic kidney disease-free survival stratified by the causative gene of Kabuki syndrome (A), genotype of *KMT2D* variants (B), and variant location of predicated loss-of-function (pLoF) variants in *KMT2D* (C).
